# A case of acute interstitial nephritis superimposed on rhabdomyolysis in a refugee patient

**DOI:** 10.1007/s40620-024-01895-x

**Published:** 2024-02-01

**Authors:** Ahmet Murt, Elif Itir Sen, Eren Altun

**Affiliations:** 1 Nephrology Clinic, Bagcilar Training and Research Hospital, Istanbul, Turkey; 2 Department of Internal Medicine, Bagcilar Training and Research Hospital, Istanbul, Turkey; 3 Department of Pathology, Bagcilar Training and Research Hospital, Istanbul, Turkey

**Keywords:** Refugee, Acute kidney injury, Rhabdomyolysis, Acute interstitial nephritis, Case report

## The case

A 46-year-old male patient of Syrian origin, who has temporary protection status in Turkey, was brought to our Emergency Department with superficial wounds all over his body. He had nausea, malaise, swollen extremities and muscle weakness. He was tachypneic at rest. His blood pressure was 155/90 mmHg. Laboratory work-up showed a serum creatinine level of 10.69 mg/dL and he had hyperkalemia (6.11 mmol/L). He had a creatine kinase level of 14,117 U/L and high transaminases. As he also had acidosis with a bicarbonate level of 14 mmol/L and oliguria (150 cc in the previous 6 h), urgent hemodialysis was prescribed. He was then admitted to our nephrology ward. Other laboratory values on admission can be found in Table 1.

**Table 1:** Laboratory values at admission

Urea (mg/dL)	204.2
Creatinine (mg/dL)	10.69
Sodium (mmol/L)	138
Potassium (mmol/L)	6.11
Calcium (mg/dL)	7.9
Phosphorus (mg/dL)	7.49
Albumin (g/dL)	2.98
Magnesium (mg/dL)	2.08
Alanine aminotransferase (ALT) (U/L)	277
Aspartate aminotransferas (AST) (U/L)	394
Uric acid (mg/dL)	4.7
C-Reactive Protein (mg/L)	40.76
Creatine Kinase (U/L)	14,117
Lactate dehydrogenase (U/L)	997
Total bilirubin (mg/dL)	0.7
Direct bilirubin (mg/dL)	0.25
Hemoglobin (g/dL)	10.8
Platelet (*10^3^/μL)	169

Medical history was taken with the help of an interpreter. He reported no use of medications and had no medical, surgical or family history to report. He stated that he had been trying to cross the Turkish/Greek border a few days earlier, when he was spotted by the Greek patrols at the border, after which he ran for some hours towards the forest on the Turkish side and hid there. He spent three days in the forest where he had no access to food, clean water or shelter. He drank water from puddles and ate plants he thought were edible. After some time, he lost consciousness and can not remember what happened afterwards. According to the Turkish security report, he was found by villagers and taken to a nearby healthcare facility. Because of his unstable general condition, he was referred to our hospital in Istanbul, which serves as a tertiary healthcare center.

**Fig. 1:**
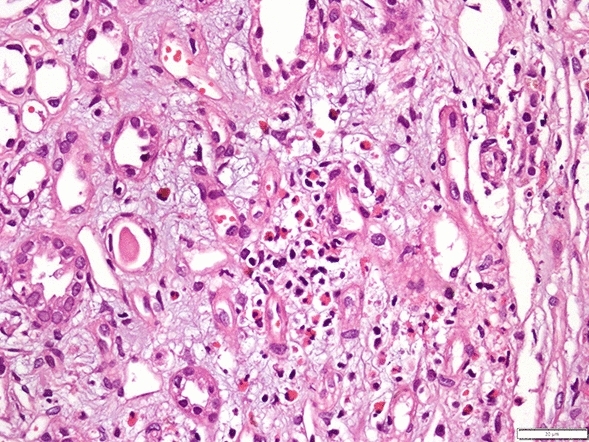
Neutrophilic and eosinophilic interstitial infiltration. Hematoxylin–Eosin staining, 40×

Based on the clinical findings and laboratory results, he was diagnosed with rhabdomyolysis. Acute kidney injury (AKI) was first attributed to rhabdomyolysis and dehydration. Following fluid therapy, creatine kinase levels gradually decreased from 14,117 to 226 U/L in 10 days. However, despite this, AKI did not resolve and he required four additional hemodialysis sessions. Further diagnostic tests were ordered: rheumatologic and vasculitis markers, including rheumatoid factor, antineutrophil antibody, anti-ds DNA, and antineutrophil cytoplasmic antibodies were all negative. Serum complement levels were normal. Hepatitis and HIV serology, as well as SARS-CoV-2 PCR were also negative. Ophthalmologic examination did not show signs of uveitis. Computed tomography of the abdomen was normal. Urine erythrocyte dipstick test was positive, and spot protein-to-creatinine ratio was 858 mg/g. In an attempt to reach a clear diagnosis, a kidney biopsy was performed on the 10th day of hospitalization, that revealed acute interstitial nephritis with eosinophilic and neutrophilic infiltrations (Fig. 1).

Following the diagnosis of acute interstitial nephritis, the patient was started on methylprednisone 1 mg/kg. On the 4th day of treatment, the patient developed polyuria and creatinine levels gradually normalized. After 10 days of methlyprednisone 1 mg/kg, we started tapering the dose, with discontinuation in 4 weeks.

## Lessons for the clinical nephrologist

Here we report the co-existence of rhabdomyolysis and acute interstitial nephritis. It was not possible to identify the exact cause of acute interstitial nephritis in this patient. Rhabdomyolysis most likely developed as a result of the patient’s long time on the run. Considering the wounds that were observed all over his body, he may have also suffered trauma. While rhabdomyolysis may cause intratubular casts, induce reactive oxygen species and tubular necrosis [1], acute interstitial nephritis due solely to rhabdomyolysis is not likely [2]. As the patient reported no use of drugs nor did he have any opportunity to get them, the agent causing acute interstitial nephritis might have been a plant, fruit or an insect bite. Another possibility is that a common insult, such as an infectious agent or a snake toxin, caused both rhabdomyolysis and acute interstitial nephritis [3, 4]. However, the clinical history was silent in this regard.

While we can not find the exact reason for concomitant rhabdomyolysis and acute interstitial nephritis in this patient, it is conceivable that the ultimate cause has to be ascribed to his refugee status. Although he felt that he had to run away and hide in the forest to save his life, this exposed him to hazardous factors. His temporary protection status in Turkey gave him the chance to receive all the necessary treatments free of charge. The patient stated that he considered himself lucky to have his kidney health back.

Turkey currently hosts the largest refugee population in the world, with an estimated 4 million individuals [5]. Although the basic healthcare needs of the refugee population are similar to those of residents, they are more vulnerable to acute diseases, mostly because they are often on the brink of survival due to poor living conditions.

Refugees in Turkey, the majority of whom come from Syria, try to cross the western borders of the country into Greece and Bulgaria to reach Europe, in the quest for a better life. This is why many try to flee across the borders or cross the Aegean Sea [6]. Unfortunately, they face additional, potentially deadly risks during their journey. The unpredictability of their situation is challenging not only for them but also for the authorities and medical staff who take care of them.

To our knowledge, this is one of the very few reports of a rather unusual presentation of AKI in a refugee patient. The underlying pathology of AKI in this patient, which involved both rhabdomyolysis and acute interstitial nephritis, may suggest considering the entity of “refugee nephropathy”, a hypothesis that needs validation, gathering further similar cases. Rhabdomyolysis may be frequent in refugees and migrants because extreme physical activity, trauma, crush syndrome, toxins and infections can result in muscular injuries, while infectious agents and toxins may cause acute interstitial nephritis. Our experience with this patient shows that both can be seen concomitantly. There are some previous examples of unusual presentation of diseases in underserved populations [7, 8].

Beyond acute diseases, caring for chronic kidney disease (CKD) in refugees is also challenging [9]. Many do not have timely access to proper care. This may increase the burden of emergent conditions for refugees with chronic kidney disease.

In conclusion, as they face poor living conditions, refugees are vulnerable to health risks. Because of their unusual exposures, they may have different disease patterns and may be simultaneously exposed to different causative factors. Nephrologists should bear in mind that different etiologic factors and disease patterns may coexist in refugee patients and they should modify their diagnostic algorithm accordingly.

## Data Availability

The data regarding this case report are available from the corresponding author upon reasonable request.
